# Quantum Dot Sensitized Photoelectrodes

**DOI:** 10.3390/nano1010079

**Published:** 2011-11-15

**Authors:** Thomas J. Macdonald, Thomas Nann

**Affiliations:** Ian Wark Research Institute, University of South Australia, Adelaide, SA 5095, Australia; E-Mail: tom.macdonald@unisa.edu.au

**Keywords:** Quantum dots, dye-sensitized photoelectrodes, photocatalysis, solar energy conversion, titania, zinc oxide

## Abstract

Quantum Dots (QDs) are promising alternatives to organic dyes as sensitisers for photocatalytic electrodes. This review article provides an overview of the current state of the art in this area. More specifically, different types of QDs with a special focus on heavy-metal free QDs and the methods for preparation and adsorption onto metal oxide electrodes (especially titania and zinc oxide) are discussed. Eventually, the key areas of necessary improvements are identified and assessed.

## 1. Introduction

Dye sensitized photoelectrodes (e.g., electrodes coated with a photocatalyst) form the basis of many exciting applications such as solar powered energy conversion (artificial photosynthesis) [1,2] self-cleaning glass [3], sterilisation [4], and others. In these applications, a dye is excited by incident light and the resulting exciton is then split at the dye/photocatalyst interface by “injection” of one charge carrier into the photocatalyst. Thus, the life-time of the charge carriers is increased and the photocatalyst is activated. The most common embodiment of this principle involves an oxide photocatalyst (for example titania (TiO_2_) or zinc oxide (ZnO)), which is sensitized by an organic or inorganic dye. A crucial component of any photocatalytic system is the dye. While most applications use organic dyes and metal organic complexes, a minority employs semiconductor nanocrystals (or so called Quantum Dots (QDs)) for the same purpose.

QDs are used because they have unique and scalable optical properties where photoluminescence and absorption of the QDs are tunable. As an example the luminescence of QDs may be tuned from the infra-red red region of the spectrum to the blue, by changing the particle size and/or composition [5]. QDs additionally provide attractive alternatives to fluorescent organic dyes because they show stable optical properties with very little or no photobleaching, have relatively narrow emission line widths and large extinction coefficients (in the order of 17,000 mol^−1^ [6]). These properties make them interesting candidates for many applications including life sciences [7], light-harvesting ‘antennas’ for solar energy conversion [8], down-converters for electro-optical devices [9], building-blocks for electronics and many other applications.

Although the above advantages sound very promising, there are three major concerns with QD sensitized photocatalysts: First, the most commonly used and commercially available QDs are all cadmium and lead based. This makes them extremely toxic and less attractive for practical use, especially in the environment. Although the production of non-toxic QDs is rapidly becoming common, they are still not as commercially available. Second, QDs have to be bound strongly onto photocatalyst films in order to obtain robust oxide based photoelectrodes for the applications mentioned above. Finally, the charge transfer kinetics between QDs and photocatalysts has to be fast in order to achieve a highly efficient photoelectrode.

In this article we review and discuss the production and properties of QD sensitized photocatalyst films. We will discuss different types of QDs that have been used to sensitise photoelectrodes and address the attachment of QDs onto these oxide films, namely TiO_2_ and ZnO. This includes a variety of approaches to obtain the most optimum surface coverage on the metal oxide photoelectrode films, to be used in photocatalytic devices.

## 2. Quantum Dots

There are a range of commercially available QDs, including CdS, CdSe, CdTe, PbS, PbSe and other class A and B elements containing semiconductors. Although these QDs are advantageous due to their optical properties, they are unacceptable for most applications particularly in environmental, medicinal and biological research due to their intrinsic toxicity. Non-toxic, high quality QDs, which can be synthesised in an economically viable way, are now paving the way for new energy related, biological and medical applications.

Cadmium and lead-based QDs are un-questionably the most studied, however it is clear that a non-toxic alternative is needed. This goal turned out to be extremely difficult to achieve. Even though some publications on InP QDs started to appear as early as 1994 [10], until recently the monodispersity and optical properties of these particles could not match those of CdSe QDs [11]. InP has band-gap characteristics similar to the cadmium materials, there are now established synthesis and characterisation methods of achieving monodisperse and highly luminescent indium-based QDs [11–14]. Significantly, these materials have the tunable photophysical attributes of the cadmium chalcogenides without their unacceptable toxicity. Fast, controlled and homogenous heating of the polar reactants tris(trimethylsilyl) phosphine (TMS3P) and indium salts avoids hot-spots and inhomogeneities such that nucleation and growth of the nanocrystals are separated, promoting the formation of monodisperse QDs. However, the synthesis of InP QDs has two significant drawbacks. TMS_3_P is a very difficult to handle reagent and cannot be purchased in many countries, and the synthesis of InP QDs requires Schlenk-techniques which cannot be scaled up easily. However, given the non-toxic nature, and robust covalent lattice of the QDs makes them very attractive for sensitising photoelectrodes, and the medical research community [15]. Furthermore, in 2010 Nann *et al.* showed InP QDs to be a suitable sensitiser for solar water splitting applications whereby they were coated with an iron catalyst forming a 3-dimensional nanophotocathode for the production of hydrogen [1]. In this work, it was possible to realise a photocathode as gold was chosen as the electrode material, as opposed to the commonly used oxide photocatalysts, which are n-type semiconductors and thus photoanodes.

CuInX_2_, CuGaX_2_, AgInX_2_, AgGaX_2_ (X = S, Se) and similar materials are alternatives to InP because they are composed of abundant, readily available, non-toxic elements (no class A and B elements present) and the use of reactive phosphine precursors is avoided. Furthermore, their optical properties cover the whole visible and near infra-red range of the spectrum [16]. CuInS_2_ QDs (CIS) have proved to be a promising compound exhibiting good light-absorbing characteristics due to them being direct band gap materials containing intrinsic highly optical absorbing coefficients [17]. The synthesis of these ternary systems is much more difficult as compared with both, IIB/VI and III/V semiconductors. The most successful synthesis method for the preparation of monodispersed nanocrystals is the hot injection method [18]. A sulfur solution is injected into a hot solution of copper and indium ions in the presence of stabilising ligands. The injection causes an immediate nucleation burst and subsequent growth of the nanocrystals. Due to the difficulty of the synthesis, first publications on the preparation of CuInX_2_ and similar QDs started to appear only recently [19,20]. However, the optical quality of these QDs was relatively low. A major break-through has been achieved by Peng *et al.*, who have published a synthesis method which results in CuInX_2_/ZnS nanoparticles with optical properties that resemble those of CdSe QDs [18]. Notably, all of the types of QDs discussed above have been used as sensitisers for photocatalytic metal oxide films. The type of the QDs affects the optical properties and performance of a photocatalytic device, where the adsorption and electronic linkage between QDs and metal oxides are governed by the surface ligands (and thus indirectly by the synthesis method) [21,22].

## 3. Photocatalytic Metal Oxides

Titania is an extensively studied and probably the most popular photocatalytic material. It is a wide-band gap semiconductor and does thus absorb ultra-violet light. Even though being very efficient, its overall ability to convert sunlight into any other form of energy is limited by its wide band-gap. Dye sensitisation extends the spectrum of light being absorbed into the visible range. The intrinsic presence of oxygen vacancies in TiO_2_ causes this metal oxide to be an n-type photoanode (the same applies to ZnO, which is the second most studied photocatalytic material).

ZnO is a semiconducting material which also has a wide-band-gap structure possessing similar properties to that of TiO_2_. The similar properties include the crystal structure, band gap and refractive index [23]. What makes ZnO a favourable material is the high electron mobility, desirable for good electron transport. This was recognised in previous literature where the typical electron mobility in ZnO was stated to be 10–100 times higher than that for TiO_2_, supporting lower electrical resistance and higher electron-transfer [24]. Although there is a considerable amount of literature on the use of ZnO semiconductors for applications in solar cells, their conversion efficiencies were reported to be 0.4–5.8% [25,26] compared with 11% for TiO_2_ [27]. It was also reported that these low efficiencies may be related to the ZnO being unstable in an acidic environment. The unstable structure was formed by an acidic dye and the Zn^2+^ ions aggregating together and/or the damage of the ZnO colloidal nanoparticles on the surface of the material [28]. The aggregation of nanoparticles makes it much harder to obtain an even surface coverage; in the following section we discuss the different adsorption methods of QDs onto metal oxide photoelectrodes.

P-type metal oxides are required in order to realise a photocathode. In 2008, Mor *et al.* generated a p-type TiO_2_ photocathode by anodisation of copper-rich titanium metal films which were co-sputtered onto fluorine-doped tin oxide (FTO) glass [29]. The conduction band of these photocathodes was found to be above the H_2_ evolution potential. However, research on p-type metal oxide semiconductors is still in its infancy [30].

## 4. Adsorption of QDs onto Metal Oxide Surfaces

There are two common approaches when attaching QDs to metal oxides, namely the chemical bath deposit (CBD) and the linker chemistry. CBD is a growth/deposition method where QDs are directly grown onto a metal oxide photocatalyst surface [31]. The other approach is using bi-functional molecules, for example carboxyl/thiol molecules as linkers [32,33]. Despite of the attachment of QDs onto metal oxide photocatalysts, surface properties such as hydrophobicity, contribution of the linker molecules to the electronic structure and electron transfer barriers contribute to the overall performance of a QD sensitized photoelectrode. Here, we focus on the linker based methods, because these methods allow for the highest flexibility of sensitising photocatalytic metal oxides and have been widely used.

Since the elemental and optical properties for QDs differ greatly upon the synthesis method, the underlying factors for surface functionalisation are the solvents and surface ligands. QDs are commonly synthesised in organic solvents such as octacedene, oleic acid, oleyamine, or trioctylphosphine (TOP). These solvents are organic and the mixtures contain many hydrophobic ligands. Therefore it can be expected that the resulting QDs are less likely to adsorb onto a hydrophilic surfaces such as TiO_2_ or ZnO. There are ways around this such as exchanging the ligands for hydrophilic ones [34]. In order to achieve hydrophilic QD surfaces, the ligand exchange methods commonly studied, all require the addition of an acidic medium. The usual exchange occurs with an excess addition of mercapto acids such as mercaptopropanoic acid (MPA) [35]. This occurs whereby the mercapto end adsorbs onto the QD surfaces by replacing the original ligands and the carboxylic end pointing into the solution. It was observed that hydrophilic QDs, adsorb onto the hydrophilic metal oxide surface (either TiO_2_ or ZnO) however there is a drawback, directly related to the now more acidic solutions these ligand exchanges create. Since metal oxides like ZnO tend to be unstable in acidic solutions (for example by dissolution or formation of hydroxides), the acidic surface ligands will react with ZnO and cause the QDs to aggregate (causing uneven coatings and difficult to achieve monolayer coverage).

Other recent studies have compared mercapto linker absorption (LA) with direct absorption (DA) [36,37]. The coating procedures for direct adsorption stay consistent throughout literature whereby the metal oxide is soaked in the QD solution, sometimes ranging from a few seconds to a few hours. Some studies even employed heating to aid their direct attachments [38]. Linker adsorption is carried out using the same way as direct adsorption; however bi-functional linker molecules like MPA are introduced. The studies illustrated that after comparing direct deposit with bi-functional ligand adsorption (using mercapto acids), the direct adsorption leads to a higher degree of QD aggregation [37]. As mentioned before, the aggregation has been thought to be due to decrease in pH, forming a more acidic solution reacting with the metal oxide surface. By addition of the base tetramethyl ammonium hydroxide (TMAH), MPA ligand exchange and hydrophilicity of the QDs is further fostered [39]. Recently, we have found that excess addition of this base increases the stability of the QDs in a ZnO metal oxide electrode but does not solve the aggregation problem. This suggests that the pH of the solution is not the only factor causing aggregation. We hypothesise that reaction of the mercapto acid with ZnO depletes the solution of ligand, which then causes aggregation of the QDs.

**Figure 1.**
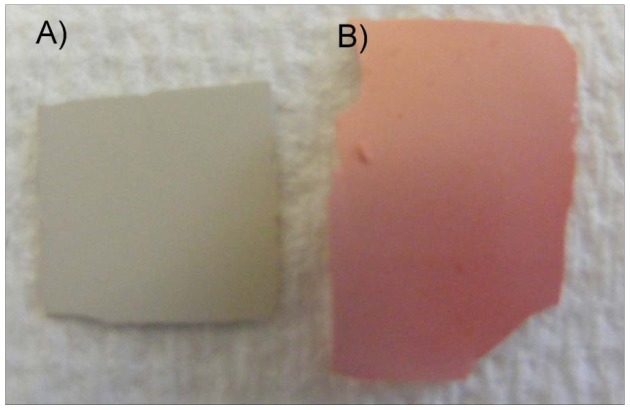
(**A**) Bare nanoporous ZnO electrode. (**B**) CdTe coated ZnO electrode. Quantum Dots (QDs) have been synthesised in aqueous solution with MPA ligands.

Recently, we were able to observe very good adsorption of aqueous CdTe QDs onto ZnO photoelectrodes (cf. Figure 1). This was indicated by a deep colour change on the photoelectrodes from white to pink.

**Figure 2.**
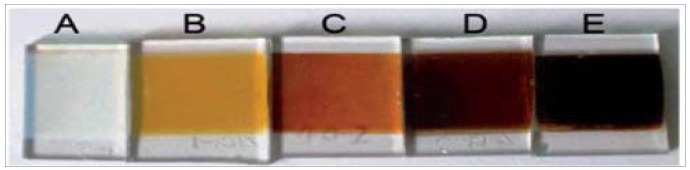
CIS QDs adsorbed onto TiO_2_ films, (**A**) a bare TiO_2_ film (**B–E**) QD/TiO_2_ film after heating for 0 s, 30 s, 80 s, and 120 s respectively (Reprinted from [40], by permission of The Royal Society of Chemistry).

Although it would be much more convenient to synthesise the QDs in water or discard the use of excess hydrophobic ligands and thiols, the quality and the stability of the QDs are better when synthesised in organic solution. The thiol ligands provide strong assembly and capping for the QDs while they remain highly dispersible in organic solvents such as chloroform and toluene. While there has been some work on the synthesis of thiol free CIS QDs aiding the ligand exchange, they are still synthesised in an organic solvent making them hydrophobic and not readily adsorbed onto a hydrophilic surface [17]. Recently, Meng *et al.* have reported strong adsorbing aqueous CIS QDs, where the advantage of this was that they were synthesised in an aqueous solution making the QD suspension completely hydrophilic [40]. This hydrophilic dispersion has been shown (Figure 2) to readily adsorb onto porous TiO_2_ photoelectrodes due to its water based solvent. These structures offer long term stability, low toxicity and show excellent photocatalytic performance in solar cells [41].

ZnO is still thought of as an alternative to use over TiO_2_ because of its ease of crystallisation and anisotropic growth along with its desirable electronic properties [28]. Since ZnO is a semiconducting film it is necessary to compare the pure film against a coated QD ZnO film. This experiment was undertaken by Chen in 2010 by using one-dimensional ZnO nanowires. They report that ZnO nanowires loaded with QDs (24 h deposition time) showed a photocurrent three times larger than that of pristine ZnO nanowires. The tests were completed using nanowires of a similar thickness (ca. 0.7 mA cm^−1^) indicating that QDs harvest light at a greater efficiency than pure ZnO nanowires [24]. Photo conversion efficiency was quantitatively reported to be 1.83%, setting the highest efficiency reported from QDs on ZnO. The current literature clearly shows that QDs require hydrophilic surface coatings in order to adsorb onto metal oxide surfaces. However, the acidity and reactivity of common ligands is a major problem for ZnO photocatalysts. This dilemma may be solved by exploring new hydrophilic linker molecules that do not affect ZnO surfaces in the future.

## 5. QDs *versus* Organic Dyes as Photosensitisers

Quantum dot and organic dye sensitized nanoporous films have increasingly become the focal point for creating an ideal photocatalytic device. TiO_2_ and ZnO are two of the most common materials to be used for nanoparticle adsorption due to their proven photocatalytic activity. Thus far, the most efficient dye-sensitized solar cell was reported by O'Regan and Grätzel in 1991 where they reported exceptionally high efficiencies for the conversion of incident photons to electrical current of greater than 80%, giving an overall cell efficiency of 11% using a ruthenium based organic dye [27]. This limits the cells light harvesting ability due to the dye complex containing a relatively low extinction coefficient and having the possibility of electron recombination during the charge transfer process. Furthermore, organic dyes are intrinsically prone to photooxidation when exposed to heat and light, making it difficult to produce highly stable cells that are robust. Another disadvantage of organic dyes is that only few of them can absorb a broad spectral range, making this an issue when trying to absorb the whole solar spectrum.

Given these disadvantages and the improved optical properties of QDs, one would expect QD sensitized photocatalysts to be superior. So far, this has not been the case and we attribute the inferior properties of QD-sensitized devices to the difficulties in preparation discussed above. This problem was recognised by Zaban and Oron in 2011 who sought to widen the absorption spectra of dyes as well as improve the efficiency of the current dye-sensitized photovoltaic cells [8]. The design incorporated both the use of organic dyes as well as QDs working simultaneously as sensitisers for a photovoltaic cell. The QD antennas transferred the energy of the absorbed light to the dye molecules (most likely by Förster Resonance Energy Transfer (FRET)) resulting in a highly efficient energy transfer and improved performance of the device. Exchanging the use of organic dyes for QDs or using a combination of both therefore shows optimisation of light harvesting for use in photovoltaic devices.

## 6. Conclusions

The superior optical properties of QDs make them clearly interesting candidates as dyes for dye-sensitized photoelectrodes. Compared with organic dyes, they show better optical stability, large extinction coefficients and adaptability to the solar spectrum. A major drawback has always been the fact that almost any high-quality QDs comprised of highly toxic elements. However, the recent advent of less-toxic alternatives like InP or CIS seems to solve this problem. Given these circumstances, it is surprising that the most efficient photocatalytic electrodes use organic sensitisers so far. This leads to the conclusion that the methods for sensitising the underlying metal oxide photocatalysts still have a high potential for improvement.

It has been shown that QDs need to possess hydrophilic surface properties in order to adsorb onto metal oxide surfaces efficiently (QDs with hydrophobic surface ligands do adsorb to some degree, but not as readily as hydrophilic ones). There are two major strategies to obtain hydrophilic QDs: first, synthesis of QDs in aqueous solution; second, synthesis of QDs in organic solution with subsequent ligand exchange. Both methods lead to similar results.

The two most important photocatalytic metal oxides are titania and zinc oxide. Even though their physical properties are very similar, it has been observed that QD adsorption shows some distinct differences. Especially with zinc oxide, typical surface ligands such as MPA react with the oxide surface and influence the QD adsorption and may disrupt the heterojunction between QDs and zinc oxide. However, these phenomena have not been studied thoroughly yet.

It can be concluded that QDs are promising sensitisers for photocatalytic applications; however, current sensitisation methods do not lead to a satisfactory heterojunction between QDs and metal oxides. This is most likely to do with the surface ligands of the QDs as noticed by several authors already. Future work is necessary to investigate the interaction of QD surface ligands with metal oxides, the QD/metal oxide heterojunction and, in particular, the influence of the QD surface ligands on the electronic structure of the metal oxide surface and heterojunction.
